# 

**Published:** 1915-03-27

**Authors:** 


### Medical Appointments.

Mr. John Morley, Ch.M., F.R.C.S., at present serving as a lieutenant in the Royal Army Medical Corps (T.F.) at the British Military Hospital, Khartoum, Sudan, has been appointed an honorary visiting surgeon to the Manchester Children's Hospital.

Dr. Henry Gott, M.B., B.Sc. Durham, M.R.C.S. Eng., L.R.C.P. Lond., has been appointed by the Camberwell Board of Guardians temporary resident medical officer at the Constance Road Institution. He has been in general practice since March 1914 at Pontyberon, and was previously resident medical officer at the Battersea General Hospital and house surgeon at Stafford County Hospital. He takes the place of Dr. Martin A. Kirton, who was appointed on the 4th inst., but four days later he informed the Board that he had been called up for service with the R.A.M.C. Dr. G. W. FitzHenry, who a month ago was appointed medical officer for the East Dulwich district, has intimated to the Guardians that he is offering his services to the War Office.

The Metropolitan Asylums Board have sanctioned the following annual appointments:—Dr. J. Galloway, consulting physician for skin diseases at Queen Mary's Hospital and Goldie Leigh Homes, £350 per annum; Mr. P. O'C. Finigan, visiting dentist at all the children's institutions, except seaside homes, £350; Dr. C. K. Bowes, medical officer at St. Anne's Home, £100; Mr. W. G. Sutcliffe, F.R.C.S. (serving with his Majesty's Forces), medical officer at East Cliff House, £125; Dr. C. E. Last, medical officer at Millfield, £100; Mr. E. Treacher Collins, F.R.C.S., ophthalmic surgeon at the Ophthalmic Schools, £300.

Mr. George Norgate Hooper, of Beckenham, has left £105 to the Middlesex Hospital in memory of an old friend, and stated that he made no further bequests to public institutions as he preferred to make gifts in his lifetime when he could watch and exercise some control over the expenditure.

### Buildings Contemplated.

Balby.—The Doncaster Town Council have adopted plans and an estimate of £25,700 for a new isolation hospital.

Claypole.—The Rural District Council have instructed the surveyor to prepare plans for an isolation hospital.

Wigan.—Work has been commenced on the proposed new out-patient department at the infirmary, estimated to cost £12,000. Messrs. W. C. Ralph and Son, King Street, Wigan, are the architects, and Messrs. Massey Bros., of Pemberton, are the contractors.

Windermere.—The Urban District Council have decided to apply to the Public Loan Commissioners for a further advance of £3,000 for the smallpox hospital.

### Obituary.

Lieutenant Theodore Stewart Lukis, 13th (Kensington) Battalion London Regiment, has died at Boulogne Base Hospital. The eldest son of Surgeon-General Sir C. Pardey Lukis, Director-General I.M.S., he studied at St. Bartholomew's Hospital, took the London M.D. and M.R.C.P. degrees, and was appointed Demonstrator of Physiology at that hospital. His spare time was devoted to social work at Toynbee Hall and to the Boy Scout movement in Hoxton. He obtained his commission in September last. On March 13 he was wounded in the trenches.

### CONTENTS—(*continued*).

| | PAGE |
|---|---|
| **The Editor's Letter-Box** ... ... ... ... | 585 |
| Hospitals and Payment for Wounded. | |
| **Institutional Needs** ... ... ... ... ... | 585 |
| **Maternity and Mothering** ... ... ... ... | 586 |
| The Case for Day Nurseries. | |
| **London Panel Committee** ... ... ... ... | 588 |
| **Buildings Contemplated** ... ... ... ... | 588 |
| **Obituary** ... ... ... ... ... ... ... ... ... ... ... | 588 |
| **The Institutional Worker** ... ... (Suppl.) | 1 |
| Institutional Workers and their Work: II. The Assistant Secretary. | |
| Question for March. | |
| Questions Invited. | |
| Employment and Training. | |
| Miscellaneous. | |

Rates of Subscription: Twelve months, 6/6; Six months, 4/-; Three months, 2/-, post free to any address in the United Kingdom. Abroad, Twelve months, 8/8; Six months, 5/-; Three months, 2/6, post free.—Address The Subscription Dept., "The Hospital," The Hospital Building, 28/29 Southampton Street, Strand, London, W.C.

